# Heartland Virus and Hemophagocytic Lymphohistiocytosis in Immunocompromised Patient, Missouri, USA

**DOI:** 10.3201/eid2405.171802

**Published:** 2018-05

**Authors:** Abigail L. Carlson, Daniel M. Pastula, Amy J. Lambert, J. Erin Staples, Atis Muehlenbachs, George Turabelidze, Charles S. Eby, Jesse Keller, Brian Hess, Richard S. Buller, Gregory A. Storch, Kathleen Byrnes, Louis Dehner, Nigar Kirmani, F. Matthew Kuhlmann

**Affiliations:** Veterans Affairs St. Louis Health Care System, St. Louis, Missouri, USA (A.L. Carlson);; Washington University School of Medicine, St. Louis (A.L. Carlson, C.S. Eby, J. Keller, B. Hess, R.S. Buller, G.A. Storch, K. Byrnes, L. Dehner, N. Kirmani, F.M. Kuhlmann);; Centers for Disease Control and Prevention, Fort Collins, Colorado, USA (D.M. Pastula, A.J. Lambert, J.E. Staples);; Centers for Disease Control and Prevention, Atlanta, Georgia, USA (D.M. Pastula, A. Muehlenbachs);; Missouri Department of Health and Senior Services, Jefferson City, Missouri, USA (G. Turabelidze)

**Keywords:** heartland virus, arbovirus, Bunyaviridae, Phlebovirus, Phenuiviridae, Hantaviridae Missouri, United States, hemophagocytic lymphohistiocytosis, immunocompromised, viruses

## Abstract

Heartland virus is a suspected tickborne pathogen in the United States. We describe a case of hemophagocytic lymphohistiocytosis, then death, in an immunosuppressed elderly man in Missouri, USA, who was infected with Heartland virus.

Heartland virus (HRTV; genus *Phlebovirus*, family *Phenuiviridae* **[previously *Bunyaviridae*]**) is a suspected tickborne pathogen in the United States (*1*). The virus was initially identified in 2009, and 9 cases of HRTV disease have been reported in the literature (*2*–*5*). Despite common features, the full spectrum of illness is unknown. We describe a fatal case of HRTV infection with hemophagocytic lymphohistiocytosis (HLH).

## The Case

An elderly man from central Missouri, USA, came to the emergency department of a local hospital in June (year redacted) reporting 6 days of nausea, anorexia, and fatigue, followed by confusion and shortness of breath with cough. He denied fever, chills, or chest pain. He worked outdoors and had numerous tick exposures. His medical history included diabetes mellitus, chronic obstructive pulmonary disease, hypertension, coronary artery disease with ischemic cardiomyopathy, hypothyroidism, and rheumatoid arthritis; he was taking prednisone, methotrexate, and adalimumab.

On initial examination, he was afebrile (36.6°C), oriented only to year, and wheezed bilaterally on expiration. Laboratory results (Table 1) showed acute kidney injury, transaminitis, and mixed anion-gap metabolic acidosis and respiratory alkalosis. Initial complete blood count results showed normocytic anemia and thrombocytopenia. Total leukocyte count was within reference range, but lymphocyte count showed absolute lymphopenia. Troponin I was mildly elevated without electrocardiographic changes. Results of chest radiography and noncontrast computed tomography of the head were unremarkable. He was transferred to a tertiary care center for management of possible acute coronary syndrome and exacerbation of chronic obstructive pulmonary disease.

**Table 1:** Selected laboratory values of immunocompromised patient leading to diagnoses of Heartland virus and hemophagocytic lymphohistiocytosis, Missouri, USA*

Test type	Reference range	4 mo before symptom onset	Post–symptom onset day				
			6	8	11	18	20
Leukocyte count, × 10^3^ cells/µL	3.8–9.8	13.7	5.8	5.00	1.60	0.2	NR
Absolute neutrophil count, cells/µL	1,800–6,600	7,400	5,000	4,100	1,200	<100	NR
Absolute lymphocyte count, cells/µL	1,200–3,300	3,300	700	500	400	100	NR
Hemoglobin, g/dL	13.8–17.2	12.1	11.8	10.5	7.1	7.0	NR
Hematocrit, %	40.7–50.3	36.4	32.5	30.5	21.2	21.2	NR
Platelets, × 10^3^/µL	140–440	202	76	42	47	19	NR
International normalized ratio	0.90–1.20	1.06	1.0	1.19	1.15	1.47	NR
Partial thromboplastin time, s	25.0–37.0	46	40	53.1	56.6	39.7	38.3
Lactate dehydrogenase, units/L	100–250	NR	422	641	3040	NR	NR
Haptoglobin, mg/dL	27–220	NR	NR	208	227	NR	NR
Ferritin, ng/mL	22–322	NR	NR	6,308	53,666	NR	NR
Fibrinogen, mg/dL	170–400	NR	NR	NR	215	NR	NR
Sodium, mmol/L	135–145	139	128	141	141	138	136
Potassium, mmol/L	3.3–4.9	4.0	5.1	5.9	5.1	5.5	4.8
Carbon dioxide, mmol/L	22–32	27	13	15	16	21	25
Blood urea nitrogen, mg/dL	8–25	16	90	63	94	60	50
Creatinine, mg/dL	0.70–1.30	1.31	3.38	1.75	4.74	1.95†	1.80†
Troponin I, ng/mL	0.00–0.03	2.55	0.76	0.27	NR	NR	NR
Cholesterol, total, mg/dL	0–200	258	115	NR	NR	NR	NR
Triglycerides, mg/dL	0–150	426	532	NR	NR	NR	NR
Aspartate aminotransferase, units/L	11–47	32	231	147	684	NR	146
Alanine aminotransferase, units/L	7–53	17	186	112	118	NR	52
Alkaline phosphatase, units/L	38–126	85	60	65	111	NR	65
Bilirubin, total, mg/dL	0.3–1.1	0.3	0.1	0.2	0.2	NR	0.7
Bilirubin, direct, mg/dL	0.0–0.3	0.1	0.1	NR	0.2	NR	NR
Amylase, units/L	28–100	NR	234	NR	NR	NR	NR
Lipase, units/L	0–99	NR	578	NR	NR	NR	NR
pH	7.35–7.45	NR	7.31	7.17	7.24	7.42	7.32
P_a_CO_2_, mm Hg	35–45	NR	21	41	38	36	47
P_a_O_2_, mm Hg	80–105	NR	125	93	108	159	96
Temperature, °C	35.5–38.3	NR	36.6	38.9	38.2	37.3	35.4
F_i_O_2_	0.21	NR	0.40	0.40	NR	0.40	NR

*F_i_O_2_, fraction of inspired oxygen; NR, not reported; P_a_CO_2_, arterial partial pressure of carbon dioxide; P_a_O_2_, arterial partial pressure of oxygen. †On continuous venovenous hemodialysis.

**Figure 1:**
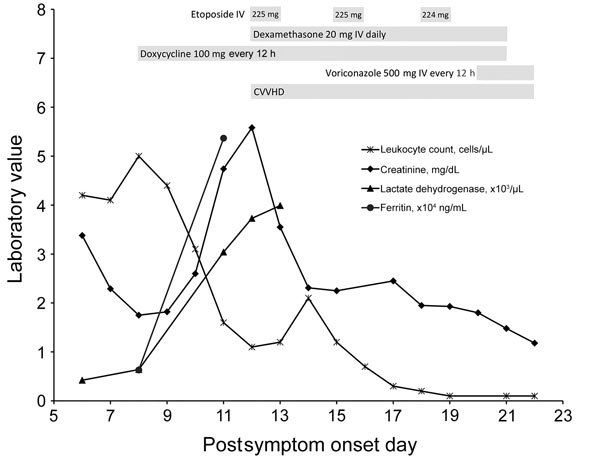
Chronology of selected laboratory findings and therapeutic interventions for immunocompromised patient infected with Heartland virus, Missouri, USA. Gray bars indicate treatments administered. CVVHD, continuous veno-venous hemodialysis; IV, intravenous.

On post–symptom onset day (PSOD) 8, the patient became febrile (38.9°C) and increasingly confused; we intubated him for airway protection. We empirically prescribed vancomycin, meropenem, ampicillin, and acyclovir for meningoencephalitis, as well as doxycycline for possible ehrlichiosis (Figure 1). We administered a platelet transfusion to complete a lumbar puncture safely. Lumbar puncture results revealed a mildly elevated cerebrospinal fluid (CSF) protein of 56 mg/dL and an unremarkable CSF glucose level of 64 mg/dL. Specimen tubes 1 and 4 cell counts were, respectively, 14 and 0 leukocytes/µL and 158 and 14 red blood cells/µL.

Initial testing for an infectious etiology of the illness was negative (Table 2), including a low positive rickettsia IgG titer, for which repeated testing was negative. Chest radiograph on PSOD 11 showed new multifocal infiltrates; a tracheal aspirate grew *Stenotrophomonas maltophilia* in culture, and we started levofloxacin. On the same day, we documented leukopenia, and a core bone marrow biopsy demonstrated hypocellularity for his age without blasts, dysplasia, or atypia. We were unable to obtain an aspirate sample. We suspected HLH; his ferritin had increased from 6,308 ng/mL on PSOD 8 to 53,666 ng/mL on PSOD 11 (reference 22–322 ng/dL). In addition, he had fever, leukopenia, thrombocytopenia, and hypertriglyceridemia (Table 1), meeting at that time 4 of 5 required diagnostic criteria by the HLH-2004 Histiocyte Society guidelines (*6*). We initiated presumptive HLH treatment with etoposide and high-dose dexamethasone on PSOD 12. We stopped vancomycin and meropenem on PSOD 18 but restarted on PSOD 20 to treat suspected sepsis after hypothermia and hypotension developed. The same day, we started voriconazole therapy to treat the patient for *Aspergillus terreus* identified from a sputum culture taken on PSOD 9. *A. terreus* had been deemed a contaminant, but we subsequently chose to treat it as a pathogen because of the patient’s leukopenia and respiratory failure. On PSOD 20, the Centers for Disease Control and Prevention (Fort Collins, CO, USA) notified the clinical care team that a blood sample obtained on PSOD 14 was positive for HRTV RNA) by reverse transcription PCR (RT-PCR) and positive for HRTV neutralizing antibodies by plaque reduction neutralization test (titer 10). Because of the patient’s clinical decline, his family elected to transition to comfort care, and he died on PSOD 22.

**Table 2:** Infectious disease testing of immunocompromised patient after symptom onset, Missouri, USA*

PSOD	Test and sample type	Result
6	Aerobic culture, urine	Nonsignificant growth
7	*Rickettsia* SFG IgG, serum	**1:64 (normal <1:64)**
	*Rickettsia* SFG IgM, serum	<1:64 (normal <1:64)
	HIV 1, 2 antibody, serum	Negative
	Epstein-Barr viral capsid antibody, IgM, serum	Nonreactive
8	Aerobic and anaerobic culture, blood × 2	No growth
	*Ehrlichia* and *Anaplasma* PCR, blood	Negative
	Enterovirus RT-PCR, CSF	Negative
	Cytomegalovirus PCR, CSF	Negative
	West Nile virus IgG, CSF	Negative
	West Nile virus IgM, CSF	Negative
	Cryptococcal antigen, CSF	Negative
	Fungal culture, CSF	No growth
	Aerobic culture, CSF	No growth
	Fungal culture, blood	No growth
9	Aerobic culture, tracheal aspirate	***Aspergillus terreus***
	Aerobic culture, urine	No growth
	Aerobic and anaerobic culture, blood × 2	No growth
10	Aerobic and anaerobic culture, blood × 2	No growth
	Acid-fast bacilli culture, blood	No growth
	Fungal culture, blood	No growth
	*Ehrlichia* and *Anaplasma* PCR, blood	Negative
	Cytomegalovirus PCR, blood	Not detected
	*Histoplasma* antigen, urine	Negative
	*Aspergillus *galactomannan antigen, blood	Negative
	*Rickettsia* SFG IgG, serum	<1:64 (normal <1:64)
	*Rickettsia* SFG IgM, serum	<1:64 (normal <1:64)
11	Aerobic culture, tracheal aspirate	**≥100,000 colonies/mL *Stenotrophomonas maltophilia*; ≥100,000 colonies/mL yeast**
14	Heartland virus RT-PCR, blood	**Positive**
20	Aerobic and anaerobic culture, blood	***Candida albicans***
	Fungal culture, blood	No growth
	Cytomegalovirus PCR, blood	Not detected
Autopsy	Heartland virus RT-PCR, blood	**Positive**
	Heartland virus RT-PCR, lymph node	**Positive**
	Heartland virus RT-PCR, spleen	**Positive**

*Positive findings are in boldface type. CSF, cerebrospinal fluid; HIV, human immunodeficiency virus; PSOD, post–symptom onset day; RT-PCR, reverse transcription PCR; SFG, spotted fever group.

**Figure 2:**
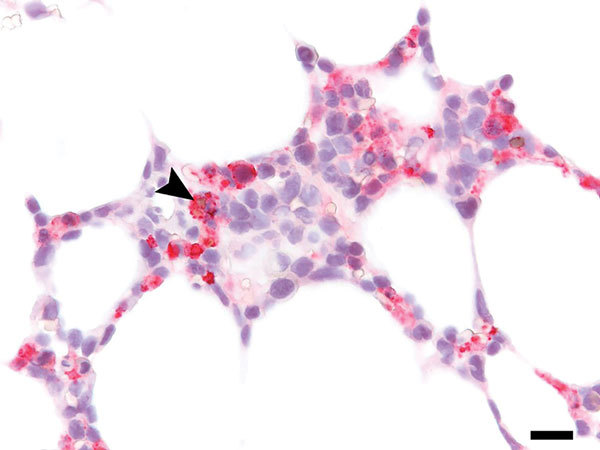
Immunohistochemistry of bone marrow from immunocompromised patient infected with Heartland virus (HRTV), Missouri, USA. Testing of biopsied sample from post–symptom onset day 11 shows extensive positive staining for HRTV antigen, including erythrophagocytosis by an HRTV-antigen–positive cell (arrowhead). Scale bar indicates 20μm.

Autopsy revealed splenomegaly and erythrophagocytosis with histiocytic hyperplasia in bone marrow, spleen, and lymph nodes, consistent with HLH. In addition, disseminated angioinvasive candidiasis was seen, and *Candida albicans* was isolated from blood cultures previously taken on PSOD 20. Central nervous system (CNS) findings included multiple brain infarcts without evidence of meningitis or encephalitis. Grocott’s methenamine silver stains of the occipital lobe were negative for yeast. All autopsy tissues were negative by HRTV immunohistochemistry (IHC) performed as previously described (*4*). However, the earlier bone marrow core biopsy had extensive HRTV antigen identified by retrospectively performed IHC (Figure 2). Autopsy specimens of blood, lymph nodes, and spleen were positive for HRTV RNA by RT-PCR.

The CSF (tube 4) and blood samples obtained on PSOD 8 were analyzed retrospectively for HRTV by using real-time PCR assay primers and probes as previously described (*3*). HRTV RNA was detected in both specimens, although at substantively higher levels in the blood (cycle threshold 20) than in the CSF (cycle threshold 32).

## Conclusions

HRTV was first identified in 2009, when 2 Missouri farmers who had been bitten by ticks were admitted to a hospital for fever, fatigue, and anorexia (*3*). Since then, descriptions of >7 additional cases, including 2 deaths, have been published (*2*,*4*,*5*). HRTV is believed to be transmitted by the lone star tick (*Amblyomma americanum*) and may be present in various mammals (*7*–*9*). This patient’s condition was similar to those described in the literature, who had fatigue, anorexia, thrombocytopenia, and transaminitis at hospital admission.

HLH is a syndrome of T-cell and macrophage hyperactivation, leading to elevated cytokines and end-organ dysfunction (*10*). Secondary HLH is often precipitated by infection, although malignancy and autoimmune diseases are also common precipitants. The HLH-2004 Histiocyte Society guidelines provide 8 diagnostic criteria for the syndrome, 5 of which must be met to establish the diagnosis (*6*). However, these guidelines were written on the basis of pediatric case series, and controversy remains regarding their sensitivity, specificity, and applicability in adults with HLH (*11*–*13*). We identified 4 HLH criteria at the time of treatment: fever, bicytopenia, hypertriglyceridemia, and hyperferritinemia. Two additional criteria, splenomegaly and hemophagocytosis, were documented at autopsy. Tests were not done for natural killer cell activity or soluble CD25 receptor levels.

We cannot directly prove that HRTV infection led to HLH in this case; however, there is a probable association. First, 4 HLH criteria were met on PSOD 8, before the identification of other infections (e.g., *S. maltophilia* pneumonia and candidemia), although these conditions may have contributed to the HLH clinical course once present. Second, HRTV without *Candida* spp. was detectable in the bone marrow at the time HLH was diagnosed, and erythrophagocytosis by HRTV antigen–positive cells in bone marrow were seen in the retrospective IHC analysis (Figure 2). Finally, 1 prior HRTV case report also detected hemophagocytosis in a lymph node (*4*).

This patient’s severe disseminated HRTV infection may have been exacerbated by his immunosuppressant medications, co-infections, or underlying conditions and could have been further exacerbated by etoposide and dexamethasone treatment. Multiple underlying conditions were also noted in another reported patient with fatal HRTV disease (*4*). We detected HRTV RNA in this patient’s CSF by RT-PCR, which may reflect CNS dissemination or may be from contamination with blood during the lumbar puncture. Further investigation is necessary to determine if HRTV can invade the CNS.

Increasing recognition of HRTV disease will support generating further data on clinical characteristics of and risk factors for higher severity. Clinicians should be alert to the possibility of severe HRTV disease, including the potential development of HLH, in persons who are immunosuppressed, have multiple concurrent conditions, or both. Early recognition of HLH, treatment of patients diagnosed with this condition, and referral to tertiary care centers should be considered in these situations.
